# Association Between Low Handgrip Strength and 90-Day Mortality Among Older Chinese Inpatients: A National Multicenter Prospective Cohort Study

**DOI:** 10.3389/fnut.2021.628628

**Published:** 2021-06-29

**Authors:** Xiao-Ming Zhang, Jing Jiao, Chen Zhu, Na Guo, Ying Liu, Dongmei Lv, Hui Wang, Jingfen Jin, Xianxiu Wen, Shengxiu Zhao, Xinjuan Wu, Tao Xu

**Affiliations:** ^1^Department of Nursing, Chinese Academy of Medical Sciences—Peking Union Medical College, Peking Union Medical College Hospital, Beijing, China; ^2^Department of Nursing, The Second Affiliated Hospital of Harbin Medical University, Harbin, China; ^3^Department of Nursing, Tongji Hospital, Tongji Medical College, Huazhong University of Science and Technology, Wuhan, China; ^4^Department of Nursing, The Second Affiliated Hospital, Zhejiang University School of Medicine, Hangzhou, China; ^5^Department of Nursing, Sichuan Provincial People's Hospital, Chengdu, China; ^6^Department of Nursing, Qinghai Provincial People's Hospital, Xining, China; ^7^Department of Epidemiology and Statistics, Institute of Basic Medical Sciences, Chinese Academy of Medical Sciences and School of Basic Medicine, Peking Union Medical College, Beijing, China

**Keywords:** handgrip strength, older adults, mortality, inpatient, cohort study

## Abstract

**Background:** The knowledge of the association between low handgrip strength and mortality among older Chinese inpatients is limited. Given China's aging society, a great number of older adults require hospital admission.

**Objective:** To explore the association between low handgrip strength and 90-day mortality, providing evidence for clinicians to predict the risk of mortality and improve clinical outcomes for older inpatients.

**Materials and Methods:** We conducted a national multicenter cohort study with a baseline survey from October 2018 to February 2019 and followed up for 90 days to record mortality outcomes. The assessment of handgrip strength was conducted using a hand dynamometer with the cutoff (handgrip strength < 28 kg for men and < 18 kg for women) to define low handgrip strength. Multivariable logistic regression analysis was applied to explore the association between low handgrip strength and 90-day mortality.

**Results:** A total of 8,910 older Chinese inpatients [mean (SD) age, 72.39 (5.68) years; 3,750 women (42.09%)], with a prevalence of low handgrip strength, at 49.57%, were included. Compared to inpatients with normal handgrip strength, inpatients with low handgrip strength were older, had less education, more were female, had lower activities of daily living (ADL) score, had lower BMI, higher frailty, higher rates of depression, and poorer cognitive function (all *p* < 0.05). At 90 days, after adjusting for gender, age, education, frailty, depression, ADL score, malnutrition, and diagnosis, low handgrip strength was independently associated with 90-day mortality, compared to normal handgrip strength (OR = 1.64, 95% CI:1.14–2.37; *P* = 0.008). Additionally, subgroup and interaction analysis showed a significant interaction effect (*P* = 0.031) between two age groups (65–74 years older vs. ≥ 75 years old), with the OR being 3.19 (95%CI:2.07–4.93) and 1.49 (95%CI:0.87–2.55), respectively.

**Conclusion:** Older Chinese inpatients with low handgrip strength had a 1.64-fold risk of 90-day mortality, compared to those with normal handgrip strength, indicating that clinicians need to screen early for handgrip strength and recommend corresponding interventions, such as resistance training and nutrition, as a priority for older inpatients.

**Clinical Trial Registration**: Chinese Clinical Trial Registry, Identifier: ChiCTR1800017682.

## Introduction

The number of older adults is experiencing remarkable growth worldwide, meaning that the world's population is aging, especially in China. It is estimated there will be ~350 million older adults worldwide by 2050 (https://population.un.org/wpp/DataQuery). Thus, healthcare for older adults is a challenge for every country. One of the main characteristics of aging is a significant change in body composition in terms of decreased lean body mass and incremental increased fat mass (1). Older adults often experience declining skeletal muscle, which makes up the major part of lean body mass, along with existing poor muscle strength. Muscle strength is a core component in maintaining physical ability, which assists older adults in sustaining better functional status (2). Older adults often suffer from low muscle strength, mainly due to the aging mechanism, unhealthy behaviors or lifestyle, or health status/comorbidities (3, 4).

Low muscle strength has been confirmed to be strongly associated with adverse outcomes. Handgrip strength is considered a better parameter to reflect whole-body strength because it provides reliable, simple, and rapid standardized measurements. According to the European Working Group on Sarcopenia in Older People (EWGSOP), low handgrip strength is an important component in defining sarcopenia (5). It is widely reported that low handgrip strength is associated with a decline in cognitive function (6), low quality of life (7), incidence of disability, and even mortality (8).

Recently, numerous studies have reported that patients with low handgrip strength are at increased risk of mortality or cardiovascular disease (CVD) mortality in different settings, such as in the community and hospital, in various countries (9–11). However, few studies have been conducted in a Chinese population. Chua et al. (12) conducted a cohort study with 13,789 community-dwelling adults, indicating that handgrip strength was inversely associated with mortality risk, the figure of HR being 2.05 (95%CI:1.44–2.90) compared to the extreme quartiles. However, this Chinese population was from Singapore, possessing different characteristics from Mainland China residents, as Singaporeans enjoy a high level of healthcare and standard of living. Also, the Chua et al. study (12) did not perform subgroup or interaction analysis based on gender, age, frailty, or depression. Meanwhile, Zhuang et al. (13), who conducted a large-scale study of 8,267 cancer patients in China, found that low handgrip is strongly associated with an increased risk of cancer mortality. As mentioned above, these two studies explored this association for a Chinese population; however, studies examining the association between low handgrip strength and 90-day mortality in older Chinese hospitalized patients are limited. Therefore, our study aimed to investigate the association between low handgrip strength (HS < 28 kg for men and < 18 kg for women) based on The Asian Working Group for Sarcopenia (AWGS) (14) and 90-day mortality in a large-scale prospective cohort study at multiple centers in China. In addition, we have employed these associations after adjusting different covariates, and conducted a subgroup analysis to determine whether the impact of low handgrip can be different in various groups.

## Materials and Methods

### Study Design and Participants

This was a large-scale prospective cohort study, which originally explored the prevalence of frailty and associated factors, at multiple centers in China (15). Our project was approved by the ethics committee of Peking Union Medical College Hospital. In addition, we have registered our protocols in the Chinese Clinical Trial Registry (ChiCTR1800017682) (15). In general, there are three levels of hospital in China: primary hospitals, secondary hospitals, and tertiary hospitals. Primary and secondary hospitals are primary health care institutions and regional hospitals, respectively. In comparison, tertiary hospitals are large urban referral and comprehensive hospitals, with a bed capacity exceeding 500. Tertiary hospitals are responsible for providing comprehensive medical, education, and scientific research, and can serve as medical centers providing care to multiple regions. Our study was focused on older hospitalized patients in tertiary hospitals. According to the original study design (15), we used a formula to calculate sample size. Based on the previous study, the prevalence of frailty was about 25%, and a 95% confidence level with a margin of error of 10% was defined. Therefore, considering possible non-response, 1,400-1,800 inpatients were recruited for each hospital, resulting in a total of 10,000 inpatients. A baseline survey was conducted from October 2018 to February 2019.

### Sampling Methods and Study Population

The cluster sampling methods consisted of three stages. First, we chose six provinces or municipalities according to China's administrative regions (Southwest, Northeast, North, Northwest, Southcentral, and East) with simple random sampling, eventually choosing Sichuan Province, Heilongjiang Province, Hubei Province, Beijing municipality, Qinghai Province, and Zhejiang Province. The researcher then selected each tertiary hospital from the aforementioned regions by using the same simple random sampling. Finally, patients from several departments, including surgery, medicine, neurology, orthopedics, and ICU, who met the inclusion and exclusion criteria, were continuously recruited from these five hospitals. Our inclusion criteria are defined as follows: age ≥ 65 years older; volunteered to participate in this project, and signed the consent form. Older hospitalized patients were excluded if they or their caregivers could not communicate effectively with the investigators.

### Handgrip Strength Measurement

Trained investigators employ a standard procedure to measure handgrip strength. First, an older patient stands and keeps their upper body upright, holding the handgrip dynamometer naturally down, and then pressing with maximum force for 2 s, with the data recorded in this moment. Handgrip strength was measured twice with each hand. Final handgrip strength was recorded based on maximal hand strength. Low handgrip strength was defined according to the Asian Working Group for Sarcopenia (AWGS), with a figure of HS < 28 kg for men and < 18 kg for women (14).

### Covariates Definition

Inpatient demographic variables, including gender, age, education, marital status, and ethnicity were collected within 48 h after hospital admission. Meanwhile, we also investigated other important covariates—such as smoking status, alcohol use, mobility (being bedridden), vision, hearing, sleep, urinary function, primary diagnosis, and length of hospitalization—of which we defined the variables of vision, hearing, sleep, and urinary function as dysfunction when they affected normal life. Geriatric syndromes—such as depression, frailty, cognitive impairment, and nutritional status—were also assessed. The FRAIL scale was used, with five simple questions to assess five domains (fatigue, ambulation, loss of weight, resistance, and illness). Frailty was considered with a score ≥ 3 (16). We also used the Geriatric Depression Scale 15 (GDS15) score instrument to evaluate whether inpatients had depression (17). According to this instrument, when the total score is five or more, patients can be defined as having depression. For cognitive impairment, we used a Chinese version of Mini-Cog, consisting of two components: a clock drawing test and a three-item recall task (defined by scores ≤ 2) (18). Mini-Nutritional Assessment-Short Form was applied to classify the nutrition category (defined normal nutritional status 12-14; scoring from 8 to 11 points was classified as being at risk of malnutrition, while being malnourished was defined as scoring 0-7 points) (19). We also collected the Activities of Daily Living using the Barthel Index.

### Mortality Assessment

Trained investigators performed a telephone follow-up for mortality 90 days after the baseline survey. The investigators were systematically trained for this procedure to make the outcomes reliable and credible.

### Statistical Analysis

The difference between two groups, such as low handgrip strength vs. normal handgrip strength, and deceased vs. survivors, was detected by Student's *t*-test and chi-squared test or Fisher's Exact Test when the data were displayed by continuous variables (mean ± standard deviation) and categorical data (percentage), respectively. There were ~200 primary diagnoses among these patients because of the participants who were drawn from different departments, including medicine, surgery, and other departments. For data analysis, we classified these patients into five categories (heart disease, pulmonary diseases, other internal medicine diseases, surgical diseases, and cancer). A generalized additive model (GAM) and smooth curve fitting analysis were used to identify whether there was a non-linear correlation between handgrip strength and 90-day mortality. Univariate analysis and multivariable logistic regression models were also adopted to detect the association between low handgrip strength and 90-day mortality. We listed an unadjusted model and different adjusted models according to the demographic characteristics or geriatric syndromes. Finally, subgroup and interaction were adopted to identify whether the association between low handgrip strength and 90-day mortality changed in different groups. A two-sided at alpha = 0.05 was adopted for all statistical analysis and performed using SAS9.4 software (SAS Institute Inc., Cary, NC, USA).

## Results

### Demographics

**Figure 1:**
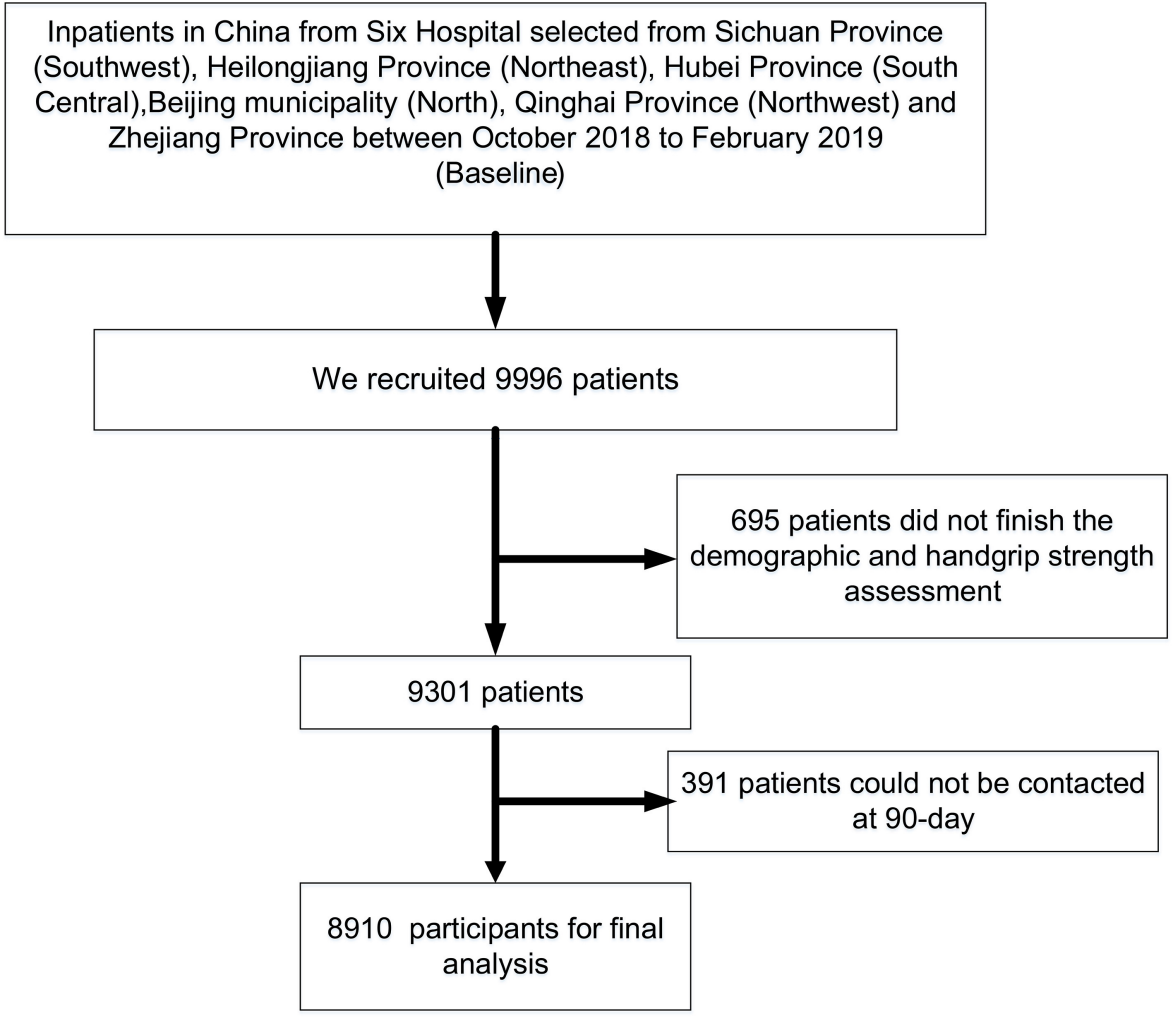
Flowchart of selction of study participants.

A total of 9,996 patients was recruited by our team at the initial stage, with 9,301 patients remaining after checking and managing the raw data due to 695 participants failing to finish the handgrip strength assessment. In addition, we eventually confirmed 8,910 patients for analysis because 391 patients could not be contacted at 90 days for the mortality assessment (Figure 1). Overall, the average age of this cohort was 72.39 (SD = 5.68) years, including 3,750 (42.09%) women. The distribution of education categories was 15.91% for illiterate, 28.66% for primary school, 40.72% for middle school, and 14.72% for university. Other detailed information is shown in Table 1.

**Table 1:** Prevalence conditions of low handgrip strength across demographics.

**Variables**	**Overall**	**Low handgrip strength**	**Normal handgrip strength**	***P*-value**
Sample size	8,910	4,417 (49.57)	4,493 (50.43)	
Age (years) (mean, SD)	72.39 ± 5.68	73.58 ± 6.07	71.22 ± 4.98	<0.0001
ADL (mean, SD)	28.00 ± 4.03	26.98 ± 4.83	28.99 ± 2.68	<0.0001
BMI (kg/m^2^) (mean, SD)	23.61 ± 3.49	23.06 ± 3.58	24.15 ± 3.31	<0.0001
Gender (*n*, %)				0.018
Female	3,750 (42.09)	1,914 (51.04)	1,836 (48.96)	
Male	5,160 (57.91)	2,503 (48.51)	2,657 (51.49)	
Ethnicity (*n*, %)				<0.0001
Han	8,376 (94.01)	4,095 (48.89)	4,281 (51.11)	
Other	534 (5.99)	322 (60.30)	212 (39.70)	
Education (*n*, %)				<0.001
Illiterate	1,417 (15.91)	904 (63.80)	513 (36.20)	
Primary school	2,553 (28.66)	1,404 (54.99)	1,149 (45.01)	
Middle school	3,628 (40.72)	1,600 (44.10)	2,028 (55.90)	
University	1,311 (14.72)	509 (38.83)	802 (61.17)	
Marital status (*n*, %)				<0.001
Marriage	7,902 (88.80)	3,826 (48.42)	4,076 (51.58)	
Divorced or widowed	997 (11.20)	584 (58.58)	413 (41.42)	
Primary diagnosis (*n*, %)				<0.001
Heart disease	1,181 (13.26)	516 (11.69)	665 (14.80)	
Pulmonary disease	6,13 (6.88)	331 (7.50)	282 (6.28)	
Other internal medicine disease	2,713 (30.46)	1,520 (34.43)	1,193 (26.55)	
Surgical disease	2,925 (32.84)	1,347 (30.51)	1,578 (17.25)	
Cancer	1,476 (16.57)	701 (15.88)	775 (35.12)	
Smoking status (*n*, %)				<0.001
Non-smoker	5,880 (65.99)	2,978 (50.65)	2,902 (49.35)	
Current smoker	991 (11.12)	427 (43.09)	564 (56.91)	
Former smoker	2,039 (22.88)	1,012 (49.63)	1,027 (50.37)	
Alcohol consumption (*n*, %)				<0.001
Non-drinker	6,805 (76.37)	3,438 (50.52)	3,367 (49.48)	
Current drinker	1,036 (11.63)	429 (41.41)	607 (58.59)	
Former drinker	1,069 (12.00)	550 (51.45)	519 (48.55)	
Bedridden (*n*, %)				<0.001
Yes	205 (2.3)	156 (76.10)	49 (23.90)	
No	8,705 (97.70)	4,261 (48.95)	4,444 (51.05)	
Vision (*n*, %)				<0.001
Dysfunction	1,943 (21.81)	1,053 (54.19)	890 (45.81)	
Normal	6,967 (78.19)	3,364 (48.28)	3,603 (51.72)	
Hearing (*n*, %)				<0.001
Dysfunction	1,674 (18.79)	948 (56.63)	726 (43.37)	
Normal	7,236 (81.21)	3,469 (47.94)	3,767 (52.06)	
Sleep (*n*, %)				<0.001
Dysfunction	3,802 (42.67)	2,049 (53.89)	1,753 (46.11)	
Normal	5,108 (57.33)	2,368 (46.36)	2,740 (53.64)	
Urinary function (*n*, %)				<0.001
Dysfunction	1,197 (13.43)	682 (56.98)	515 (43.02)	
Normal	7,713 (86.57)	3,735 (48.42)	3,978 (51.58)	
Depression (*n*, %)				<0.001
Yes	1,369 (15.64)	920 (67.20)	449 (32.80)	
No	7,385 (84.36)	3,410 (46.17)	3,975 (53.83)	
Cognitive impairment (*n*, %)				<0.001
Yes	1,684 (19.77)	1,098 (65.20)	586 (34.80)	
No	6,832 (80.23)	3,069 (44.92)	3,763 (55.08)	
MNA-SF (*n*, %)				<0.001
Normal nutritional status	4,999 (56.11)	2,030 (40.61)	2,969 (59.39)	
At risk of malnutrition	3,044 (34.16)	1,761 (57.85)	1,283 (42.15)	
Malnourished	867 (9.73)	626 (72.20)	241 (27.80)	
Frailty (*n*, %)				<0.001
Yes	1,492 (16.75)	1,075 (72.05)	417 (27.95)	
No	7,418 (83.25)	3,342 (45.05)	4,076 (54.95)	
Length of hospital stay (days) (mean, SD)	9.15 ± 5.84	9.61 ± 5.94	8.70 ± 5.72	<0.001

*ADL, basic activities of daily living; MNA-SF, Mini-Nutritional Assessment-Short Form*.

### Comparison Between Low Handgrip Strength and Normal Handgrip Strength

Table 1 describes general characteristics and other variables between low handgrip strength and normal handgrip strength. Overall, the prevalence of low handgrip strength was 49.57%. Hospitalized patients with low handgrip strength were older, had less education, more were female, and they scored lower in terms of ADL and BMI. A higher percentage of patients with low handgrip strength had been bedridden for an extended period and suffered from vision and sleep dysfunction. In addition, low handgrip strength was associated with cognitive impairment (all *P* < 0.01), malnourishment (*P* < 0.01), frailty (*P* < 0.01), and depression (*P* < 0.01). The proportion of low handgrip strength in cancer patients was higher than in patients with heart disease and pulmonary disease (15.88 vs. 11.69 vs. 7.50%). The difference in length of hospital stay was statistically significant between those with low handgrip strength and those with normal handgrip strength (*P* < 0.01).

### Univariate Analysis

There was a statistically significant difference between survivors and those who were deceased at 90 days in terms of age, ADL, BMI, gender, smoking status, alcohol use, being bedridden for an extended period, depression, Mini Nutritional Assessment short-form (MNA-SF), frailty, and length of hospital stay, with all *P-*value being < 0.05. Additionally, the rate of 90-day mortality was higher in the low handgrip strength group compared to the normal handgrip strength group, with a significant difference (2.7 vs. 1.0%, *P* < 0.0001). However, we did not find any significant differences between these groups in terms of ethnicity, education, marital status, vision, hearing, and sleep. The highest rate of 90-day mortality (4.34%) among all disease groups was in cancer patients. Meanwhile, the rate of 90-day mortality between patients with cognitive impairment and those with normal cognitive function was very close (2.4 vs. 1.7%, *P* = 0.0492). All results are shown in Table 2.

**Table 2:** Univariate analysis results.

**Variables**	**Survivors at 90-day**	**Deceased at 90-day**	***P*-value**
Sample size	8,743	167	
Age (years) (mean, SD)	72.36 ± 5.66	73.70 ± 6.19	0.002
ADL (mean, SD)	28.03 ± 4.00	26.40 ± 4.85	<0.0001
Low handgrip strength (*n*, %)			<0.0001
Yes	4,298 (97.31)	119 (2.69)	
NO	4445 (98.93)	48 (1.07)	
BMI (kg/m^2^) (mean, SD)	23.65 ± 3.47	21.82 ± 3.72	< .0001
Gender (*n*, %)			0.004
Female	3,702 (98.72)	48 (1.28)	
Male	5,041 (97.69)	119 (2.31)	
Ethnicity (*n*, %)			0.9977
Han	8,219 (98.13)	157 (1.87)	
Other	524 (98.13)	10 (1.87)	
Education (*n*, %)			0.1309
Illiterate	1,386 (97.81)	31 (2.19)	
Primary	2,512 (98.39)	41 (1.61)	
Middle	3,550 (97.85)	78 (2.15)	
University	1,294 (98.70)	17 (1.30)	
Marital status (*n*, %)			0.2534
Marriage	7,750 (98.08)	152 (1.92)	
Divorced or widowed	983 (98.60)	14 (1.40)	
Primary diagnosis (*n*, %)			<0.001
Heart disease	1,170 (99.07)	11 (0.93)	
Pulmonary disease	594 (96.90)	19 (3.10)	
Other internalmedicine diseases	2,670 (98.42)	43 (1.58)	
Surgical diseases	2,895 (98.97)	30 (1.03)	
Cancer	1,412 (95.66)	64 (4.34)	
Smoking status (*n*, %)			0.021
Non-smoker	5,775 (98.21)	105 (1.79)	
Current smoker	980 (98.89)	11 (1.11)	
Former smoker	1,988 (97.50)	51 (2.50)	
Alcohol consumption (*n*, %)			0.034
Non-drinker	6,682 (98.19)	123 (1.81)	
Current drinker	1,022 (98.65)	14 (1.35)	
Former drinker	1,039 (97.19)	30 (2.81)	
Bedridden (*n*, %)			0.0013
Yes	195 (95.12)	10 (4.88)	
No	8,548 (98.20)	157 (1.80)	
Vision (*n*, %)			0.4033
Dysfunction	1,911 (98.35)	32 (1.65)	
Normal	6,832 (98.06)	135 (1.94)	
Hearing (*n*, %)			0.7453
Dysfunction	1,641 (98.03)	33 (1.97)	
Normal	7,102 (98.15)	134 (1.85)	
Sleep (*n*, %)			0.2216
Dysfunction	3,723 (97.92)	79 (2.08)	
Normal	5,020 (98.28)	88 (1.72)	
Urinary function (*n*, %)			0.049
Dysfunction	1,166 (97.41)	31 (2.59)	
Normal	7,577 (98.24)	136 (1.76)	
Depression (*n*, %)			<0.001
Yes	1,322 (96.57)	47 (3.43)	
No	7,265 (98.38)	120 (1.62)	
Cognitive impairment (*n*, %)			0.0492
Yes	1,643 (97.57)	41 (2.43)	
No	6,715 (98.29)	117 (1.71)	
MNA-SF (*n*, %)			<0.001
Normal nutritional status	4,962 (99.26)	37 (0.74)	
At risk of malnutrition	2,967 (97.47)	77 (2.53)	
Malnourished	814 (93.89)	53 (6.11)	
Frailty (*n*, %)			<0.001
Yes	1,428 (95.71)	64 (4.29)	
No	7,315 (98.61)	103 (1.39)	
Length of hospital stay (days) (mean, SD)	9.11 ± 5.80	11.20 ± 7.72	<0.001

*ADL, basic activities of daily living;MNA-SF, Mini-Nutritional Assessment-Short Form*.

### Non-linear Relationship Analyses

**Figure 2:**
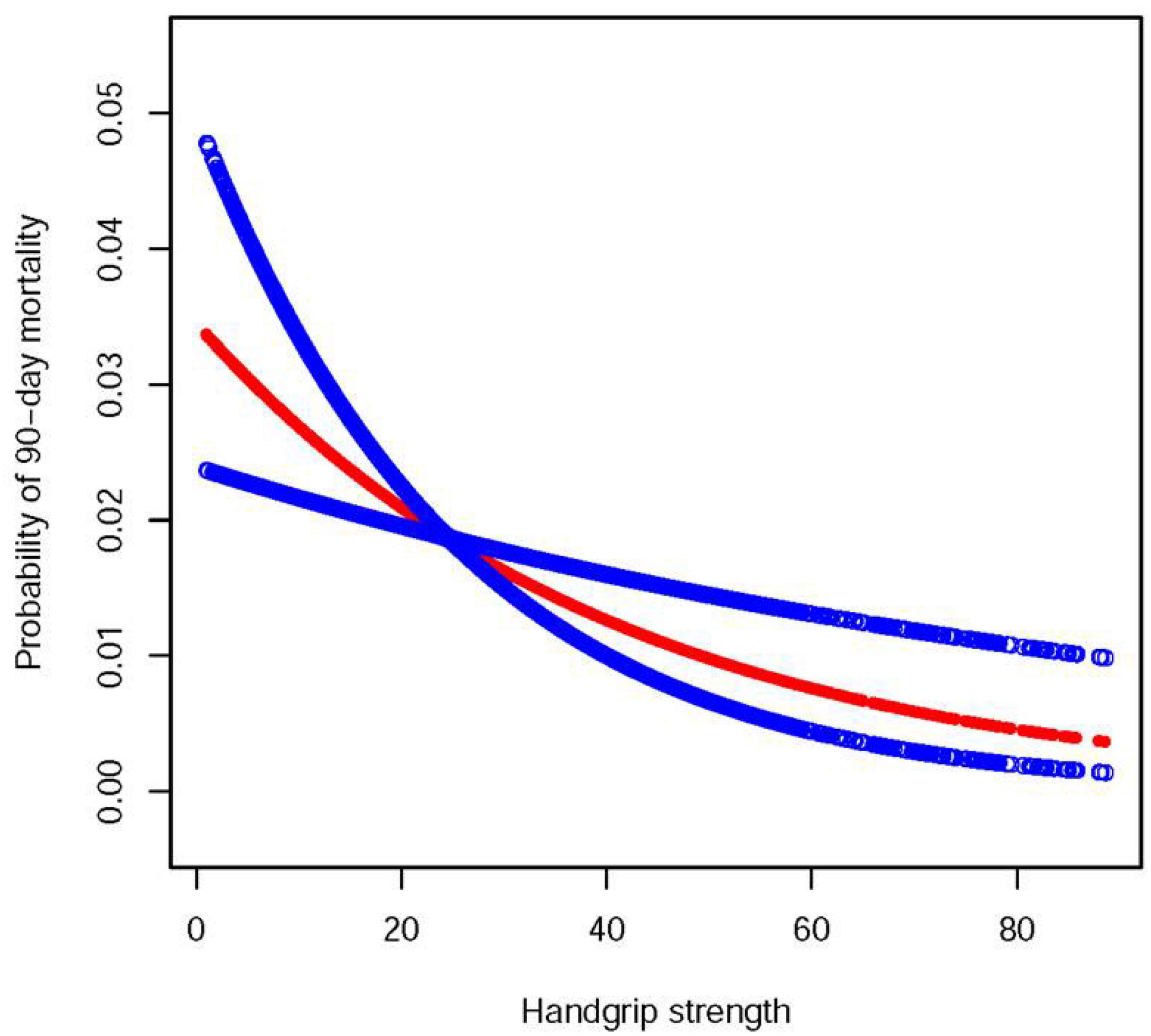
A linear relationship between handgrip strength and probability of 90-day mortality by a generalized additive model. The red dotted lines represent the estimated risk of 90-day mortality, and the blue dotted lines indicate the 95% CI of the spline plots.

A non-linear relationship between handgrip strength and 90-day mortality was performed, with the results presenting a negative linear relationship. This result indicates that with an increase in handgrip strength, the 90-day mortality rate decreased (Figure 2).

### The Relationship Between Low Handgrip Strength and 90-day Mortality

**Figure 3:**
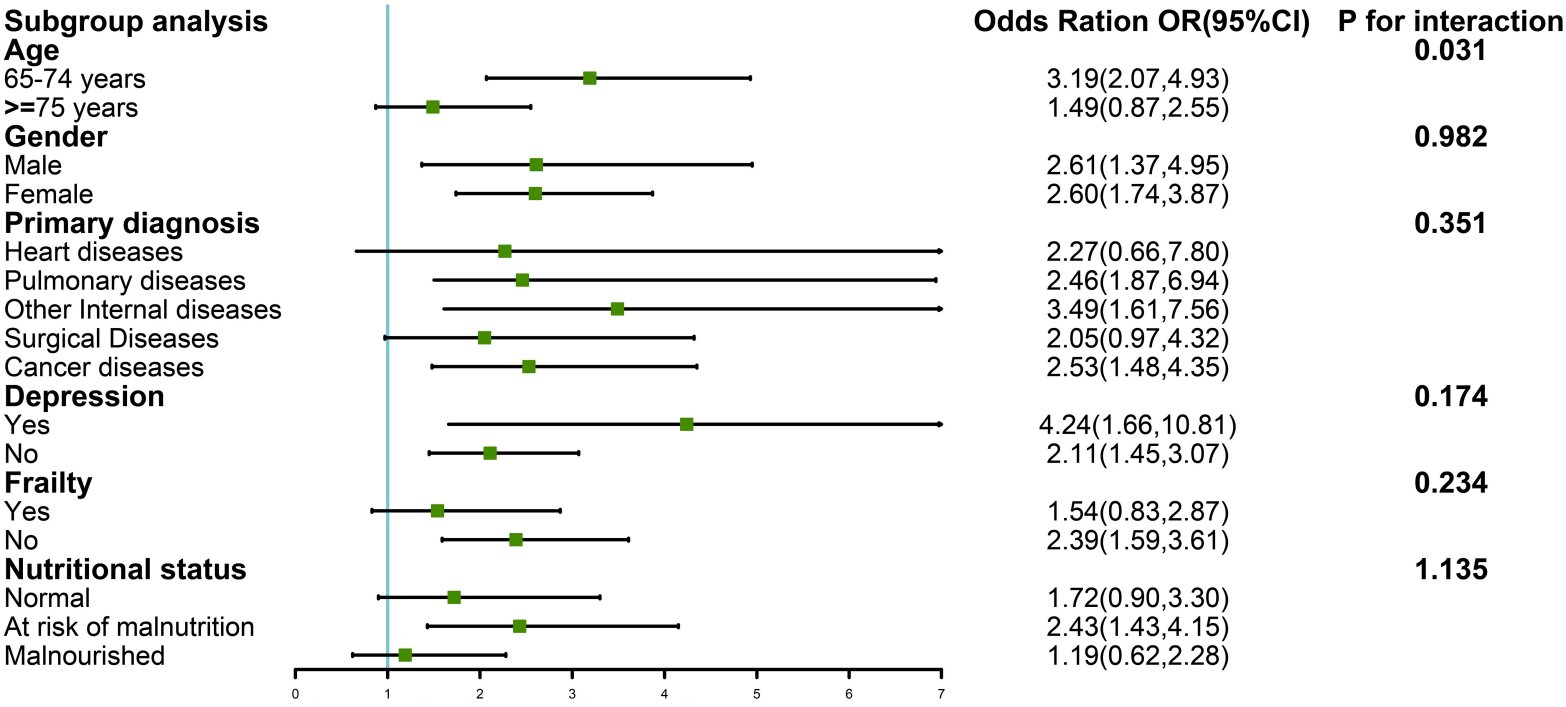
Subgroup analysis of the association between low handgrip strength and 90-day mortality.

The multivariable logistic regression analysis indicated that low handgrip strength was associated with an increased risk of 90-day mortality in an unadjusted model (OR = 2.57,95%CI:1.83–3.61; *P* < 0.001). This association was diminished when adjusting different variables. After fully adjusting for age, gender, education, frailty, depression, malnutrition, ADL, and primary diagnosis, this association remained (OR = 1.64,95%CI:1.14–2.37; *P* = 0.008). Detailed information is presented in Table 3. Furthermore, we also did a subgroup based on stratified and interaction analysis and found that the OR value of low handgrip strength and 90-day mortality was stronger in patients aged 65–74 years old than in the group aged 75 years old or more (OR = 3.19;95%CI:2.07–4.93, vs. OR = 1.49,95%CI:0.87–2.55), with a significant interaction effect (*P* = 0.031). In the other group, we did not find any significant interaction in terms of gender, depression, frailty, primary diagnosis, and MNA-SF (All *P-*value > 0.05) (Figure 3).

**Table 3:** Multivariable logistic regression analysis of the association between low handgrip strength and 90-day mortality.

**Exposure**	**Non-adjusted** ** (OR, 95%, CI)**	**Adjusted I** ** (OR, 95%, CI)**	**Adjusted II** ** (OR, 95%, CI,)**	**Adjusted III** ** (OR, 95%, CI)**	**Adjusted IV** ** (OR, 95%, CI)**
**LOWER HANDGRIP STRENGTH**					
No	Reference	Reference	Reference	Reference	Reference
Yes	2.57 (1.83-3.61); <0.001	2.43 (1.71-3.45); <0.001	2.04 (1.43-2.92); <0.001	1.97 (1.38-2.84); 0.0002	1.64 (1.14,2.37) 0.008

*Adjusted I: age gender education*.

*Adjusted II: age gender education, frailty*.

*Adjusted III: age gender education, frailty, depression*.

*Adjusted IV: age gender education, frailty, depression, ADL. Primary diagnosis*.

## Discussion

Our study indicates that older Chinese inpatients with low handgrip strength are at increased risk of 90-day mortality, compared to those with normal handgrip strength, after adjusting relevant demographic characteristics and other covariates, such as depression, frailty, ADL, primary diagnosis, and MNA-SF. It indicates that low handgrip strength could be a prognostic factor for clinicians to stratify groups at high risk of mortality. Additionally, subgroup and interaction analysis showed that this association was higher in the 65- to 74-year-old age group than in the group aged 75 years or older. This is one of the few studies, to the best of our knowledge, exploring the association between low handgrip strength and 90-day mortality in older Chinese inpatients covering various diseases.

Overall, there is a great number of studies reporting that community-dwelling older adults with low handgrip strength are at increased risk of mortality or CVD mortality. In a systematic review and meta-analysis published in 2018 (20), including 38 studies with a total of 1,907,580 participants, the findings revealed that higher handgrip strength levels were associated with a reduced risk of mortality (pooled HR = 0.69; 95% CI, 0.64–0.74). In comparison, this meta-analysis only included one study on older Chinese adults, with only 99 participants. A recent larger study on the Chinese population conducted by Zhuang et al. (13), exploring the correlation between handgrip strength (HGS) and mortality, showed that older adults with low HGS had a strongly increased risk of overall cancer mortality. In contrast, participants in our study were general inpatients and suffered from various types of disease, not exclusively from cancer. In our study, the prevalence of low handgrip strength was 49.57%, higher than in the study by Zhuang et al. (13) (25.7%). The difference may be due to the fact that the average age in our study was much higher, at 72.39 vs. 58 years old. It is reported that handgrip strength gradually declines with age (21). Thus, our study also indicates that low handgrip strength is prevalent among older Chinese inpatients.

Interestingly, we found the association between low handgrip strength and 90-day mortality was stronger in patients ages 65–74 years old than in those aged ≥ 75 years, with a significant interaction difference (*P* = 0.031). Consistent with our study, Celis-Morales et al. (22) reported that HR of the association between lower handgrip strength and all-cause mortality was higher in the younger age group than in the older age group. In addition, other studies described similar results among cancer patients (13). As we all know, the older the person, the lower the handgrip strength (23). In our study, average handgrip strength was higher in the younger age group than in the older age group (25.68 ± 12.00 kg vs. 22.01 ± 10.83 kg; *p* < 0.001). Why did we not observe that the impact of handgrip strength has a worse risk of mortality in those aged ≥ 75 years? We speculate that older inpatients have worse health, often suffering from multiple comorbidities, such as diabetes, hypertension, frailty, and cognitive impairment. Thus, these conditions conceal the effects of low handgrip strength on mortality. More studies about the age effect on low handgrip strength and mortality in older inpatients are needed to explore this question.

We found the impact of handgrip strength on 90-day mortality was similar between genders. However, in terms of the gender-specific effects of handgrip on mortality, we were unable to draw a conclusion based on the literature. Arvandi et al. (24), reported that hazard ratio (95% CI) of handgrip strength on mortality was higher in women than in men. Three studies found that low handgrip strength was not associated with mortality in women (25–27). The mechanism causing this discrepancy was unclear. Different ethnicities, hormones, study design, and sample size may have led to this inconsistent result. Thus, more large well-designed prospective cohort studies are warranted in future to examine this important issue.

Older inpatients often suffer from depression after enduring disease for an extended period. It is estimated that the prevalence of depression among inpatients ranges from 25.1 to 57.5% (28). Low handgrip strength coexisting with depression among older patients is very common (29). Our subgroup analysis results showed that the OR of mortality was higher in the group with depression than in the group without depression. However, we need to be careful that the interaction analysis of depression was not significant. One study reported by Park et al. (30) indicated that older adults with low handgrip strength coexisting with depression are at higher risk of mortality, compared to those experiencing low handgrip strength alone. The main reason may be that people suffering from depression have been reported to be at increased risk of mortality (31) and depression, which are related to low handgrip strength (32), with both factors combined leading to increased mortality risk.

The underlying mechanisms behind the association between low handgrip strength and mortality are not clearly understood, but appear to be complicated and multifactorial. First, low handgrip strength and lower muscle mass are the two main components that define sarcopenia, a condition confirmed by numerous studies to lead to a greater risk of mortality (33–36). In addition, low handgrip strength to some extent reflects decreased skeletal muscle mass due to aging, because sufficient skeletal muscle mass plays a crucial role in maintaining strength, and skeletal muscle mass is the main organ for metabolism. Aging and malnutrition lead to decreased muscle mass, a slowing metabolic rate, and reduced calorie consumption (37). This impact is intensified by inactivity, in turn giving rise to a high risk of metabolic syndrome (obesity, diabetes, dyslipidemia), eventually increasing the likelihood of mortality (38). Third, low handgrip strength can partially be explained by diminished neural system functioning, which results in poor physical function and can lead to a high risk of falls (39). As we all know, falls are one of the main reasons for a greater risk of mortality among inpatients (40). Therefore, this complicated association and mechanism warrants further exploration.

## Clinical Implications

Our study indicated that low handgrip strength could be an independent predictor for mortality among Chinese inpatients, which is consistent with other previously published studies (12, 41). Handgrip strength can be obtained by using simple equipment that is not time-consuming to use and can be applied in any clinical setting, which is very convenient for medical staff (42). In addition, according to the EWGSOP2 recommendation, muscle strength is a core component of screening for sarcopenia, because it can reflect key features of the disease, and has been reported to accompany an increased risk of adverse outcomes (5). Thus, screening inpatients for muscle strength can have multiple clinical implications. First, clinicians can stratify high-risk inpatients and initiate an intervention program—such as physical therapy, an exercise training program, and comprehensive treatment—early on, in order to reduce mortality rates. Second, clinicians can educate patients' family members to pay attention to their loved ones, for example, by suggesting a home-based resistance and aerobic exercise program to improve muscle strength.

Our study has both strengths and limitations. To the best of our knowledge, this is one of the few large sample studies of Chinese inpatients covering various types of disease based on a multiple-center prospect cohort study to explore the association between handgrip strength and 90-day mortality. In addition, we performed comprehensive analyses, such as generalized additive model (GAM), multivariable logistic regressions for adjusting variables, such as geriatric syndrome (frailty, depression, malnutrition, ADL) not controlled in previous studies, and subgroup and interaction analysis. Most importantly, our study could encourage medical staff to consider screening for handgrip strength as an important routine measurement in older hospitalized patients. At the same time, with these limitations, there must also be cautions. First, we recruited participants from different regions of China, which means that we need to be cautious about generalizing our findings to other countries. Second, we did not collect the parameter that represents muscle mass; thus, we cannot calculate sarcopenia and investigate the association between sarcopenia and 90-day mortality. Third, the follow-up length of our study was relatively short-term, which prevents us from comparing our findings to other studies that included long-term outcomes (10, 11). Fourth, although our study was a prospective cohort study, we cannot draw a definite conclusion on causal inference. Fifth, we subjectively classified these diagnoses into five groups, which might produce some bias. Sixth, the collection of data from six different tertiary hospitals; although the level of the medical service at tertiary hospitals is regulated in a standardized way in mainland China, we still cannot totally exclude the possible effect of the different hospitals themselves on our results.

## Conclusion

We reported that low handgrip strength is strongly associated with 90-day mortality among older Chinese hospitalized patients, even adjusting for demographic characteristics, depression, frailty, malnutrition, ADL, and diagnosis, indicating the usefulness of handgrip strength applied in a clinical setting for assessing and improving prognosis. Early screening and comprehensive interventions, such as nutrition and exercise programs, might be important for older hospitalized patients.

## Data Availability Statement

The raw data supporting the conclusions of this article will be made available by the authors, without undue reservation.

## Ethics Statement

The studies involving human participants were reviewed and approved by Ethics Committee of Peking Union Medical College Hospital (S-K540). Written informed consent forms to participate were obtained in the study. The patients/participants provided their written informed consent to participate in this study.

## Author Contributions

XW is responsible for designing this study and initiating this concept. X-MZ and JJiao were responsible for drafting the initial manuscript. TX conducted all statistical analyses. CZ, NG, YL, DL, HW, JJin, XW, and SZ were responsible for collecting the raw data and organizing this project. All authors contributed to the article and approved the submitted version.

## Conflict of Interest

The authors declare that the research was conducted in the absence of any commercial or financial relationships that could be construed as a potential conflict of interest.

## Abbreviations

EWGSOP: European Working Group on Sarcopenia in Older People
HR: hazard ratio
OR: odds ratio
SD: Standard deviation
BMI: Body mass index
CI: Confidence interval.
